# Advancements in markerless motion capture systems for upper limb functional assessment in stroke rehabilitation: A comprehensive review

**DOI:** 10.1177/20552076251412636

**Published:** 2026-08-03

**Authors:** Padmavathi Shenoy, S. Meenatchi Sundaram, Senthil Kumaran D, Manikandan Natarajan, Sucheta Kolekar

**Affiliations:** 1 Department of Instrumentation and Control Engineering, 125853 Manipal Institute of Technology, Manipal Academy of Higher Education, Manipal, Karnataka, India; 2 Department of Physiotherapy, 683698 Manipal College of Health Professions, Manipal Academy of Higher Education, Manipal, Karnataka, India; 3 Department of Information and Communication Technology, 125853 Manipal Institute of Technology, Manipal Academy of Higher Education, Manipal, India

**Keywords:** Markerless motion capture, stroke rehabilitation, machine learning, kinematic analysis, telerehabilitation

## Abstract

Stroke frequently leads to upper limb impairment, requiring precise evaluation to guide effective rehabilitation. Markerless motion-capture technologies have emerged as promising, noninvasive tools for objective and quantitative movement assessment. This comprehensive review summarizes advancements in markerless motion-capture systems for upper limb functional assessment in stroke rehabilitation and identifies current limitations and future opportunities. Relevant studies published between 2012 and 2025 were identified through PubMed, Scopus, IEEE Xplore, and Web of Science databases using search terms related to stroke, upper-limb motion, and markerless motion capture. Recent systems such as OpenPose, MediaPipe, and Theia3D demonstrate reliable kinematic accuracy (mean error *<*6°) and feasibility for remote rehabilitation monitoring. However, variations in study protocols, small sample sizes, and limited clinical validation continue to hinder widespread clinical adoption. Markerless motion capture provides an accessible and objective approach for assessing upper-limb function after stroke. Future work should focus on standardizing validation procedures, improving real-time performance, and integrating these systems into routine clinical and telerehabilitation workflows.

## Introduction

Stroke represents a primary contributor to global disability, profoundly affecting both the motor and cognitive functions of patients. Stroke can lead to a range of functional impairments, with reduced mobility and coordination of the upper extremities being especially significant.^1^ These impairments impede the ability to perform daily activities, thereby restricting the independence and overall quality of life for those affected. The assessment of upper limb function plays a critical role in the development of tailored rehabilitation programs, monitoring progress, and assessing treatment outcomes.^2^ Historically, assessments in this domain have depended on clinical scales, manual observations, or marker-based motion capture systems.^3^ Each of these methods presents inherent limitations, including subjectivity, limited accessibility, and complex setups.

Recent advancements in computer vision (CV) and artificial intelligence (AI) have facilitated the development of markerless, video-based techniques for motion analysis.^4^ The described methods utilize standard or depth cameras to acquire movement data, eliminating the need for subjects to wear sensors or markers The noncontact approach demonstrates reduced intrusiveness and enhancing adaptability to real-world scenarios, positioning it as a viable alternative for stroke rehabilitation.^5^ There is a high level of accuracy in figuring out joint movements and kinematic parameters with these systems because they use algorithms for pose estimation and motion tracking. The combination of video-based techniques and machine learning (ML) significantly amplifies their capabilities, facilitating automated pattern recognition, functional level classification, and rehabilitation outcome prediction. These systems significantly enhance the evaluation of critical parameters such as joint angles, trajectory smoothness, jerk, and range of motion (ROM) during the execution of specific tasks.^6,7^ The advancement of intuitive applications and devices enables healthcare professionals and patients to receive immediate feedback, promoting self-directed rehabilitation methods. Despite these advancements, challenges persist in optimizing system accuracy, ensuring reliability across diverse populations and addressing limitations such as varying lighting conditions and occlusions during motion capture.^8^ Furthermore, a significant deficiency exists in comprehensive reviews that focus on the specific components and methodologies employed in these studies. By conducting a comprehensive review of research on video-based, noncontact, markerless methods for assessing upper extremity function in poststroke rehabilitation, this study aims to bridge the gap between rapid technological advancements and their limited clinical adoption. Specifically, there is a lack of systematic reviews that examine how these technologies measure clinically relevant parameters, compare to established benchmarks and address current limitations that hinder broader implementation. This review scrutinizes the various types of cameras used, the tasks and gestures analyzed, the pose estimation methodologies, the parameters investigated, and the ML applications in this context. This analysis delineates trends, identifies limitations, and highlights opportunities, establishing a foundational framework for forthcoming advancements in stroke rehabilitation technologies.

### Background on poststroke rehabilitation

Stroke represents a predominant worldwide contributor to disability, leading to considerable motor, sensory, and cognitive deficits. The upper extremities are frequently subject to significant functional limitations, which directly hinder an individual's capacity to engage in essential daily activities, including eating, dressing, and writing.^9^ The design of poststroke rehabilitation aims to enhance functional independence, with a significant focus on the upper limbs because of their crucial role in enhancing quality of life. The recovery of upper extremity function is a multifaceted process that exhibits significant variability among individuals. Several factors influence this variability, including the severity and anatomical location of the stroke, the duration since the occurrence, and the efficacy of the implemented rehabilitation strategies. Rehabilitation programs typically encompass physical therapy,^10^ occupational therapy,^11^ and task-specific training^12^ aimed at enhancing neural plasticity and optimizing motor control. Accurate and objective evaluations of motor function significantly influence the effectiveness of these interventions, directing therapy and tracking advancements.

Conventional approaches to assessing upper limb function, including clinical scales such as the Fugl–Meyer Assessment^13^ and the Action Research Arm Test,^14^ are prevalent; however, they frequently exhibit subjectivity and rely heavily on the examiner's proficiency. Marker-based motion capture systems deliver objective data through the analysis of kinematic parameters. However, these systems necessitate specialized equipment, intricate setups, and proficient operators, which limit their accessibility for broader application. The identified limitations underscore the necessity for the development of more efficient, user-friendly, and objective assessment tools.

Recent advancements in technology, especially in the realms of CV and AI, have created new opportunities for evaluating upper extremity function through video-based, markerless methodologies. These systems utilize depth cameras in conjunction with sophisticated algorithms to monitor joint movements and assess motion, eliminating the requirement for wearable sensors. These methods facilitate noninvasive, real-time assessment of movement, equipping clinicians with comprehensive quantitative information regarding joint angles, motion smoothness, and various essential parameters.

The evaluation of upper extremity function is essential for multiple reasons. This approach facilitates the identification of distinct impairments, enables the monitoring of recovery progress over time, and allows for the customization of rehabilitation strategies to meet individual requirements. Additionally, objective assessments allow researchers and clinicians to assess the efficacy of novel therapeutic interventions, thereby facilitating the progression of stroke rehabilitation methodologies. The integration of advanced technologies into functional assessment presents opportunities to enhance accessibility, accuracy, and patient outcomes, thereby facilitating the development of more effective and personalized rehabilitation programs.

### Significance of noncontact, markerless video-based techniques

**Figure 1.**
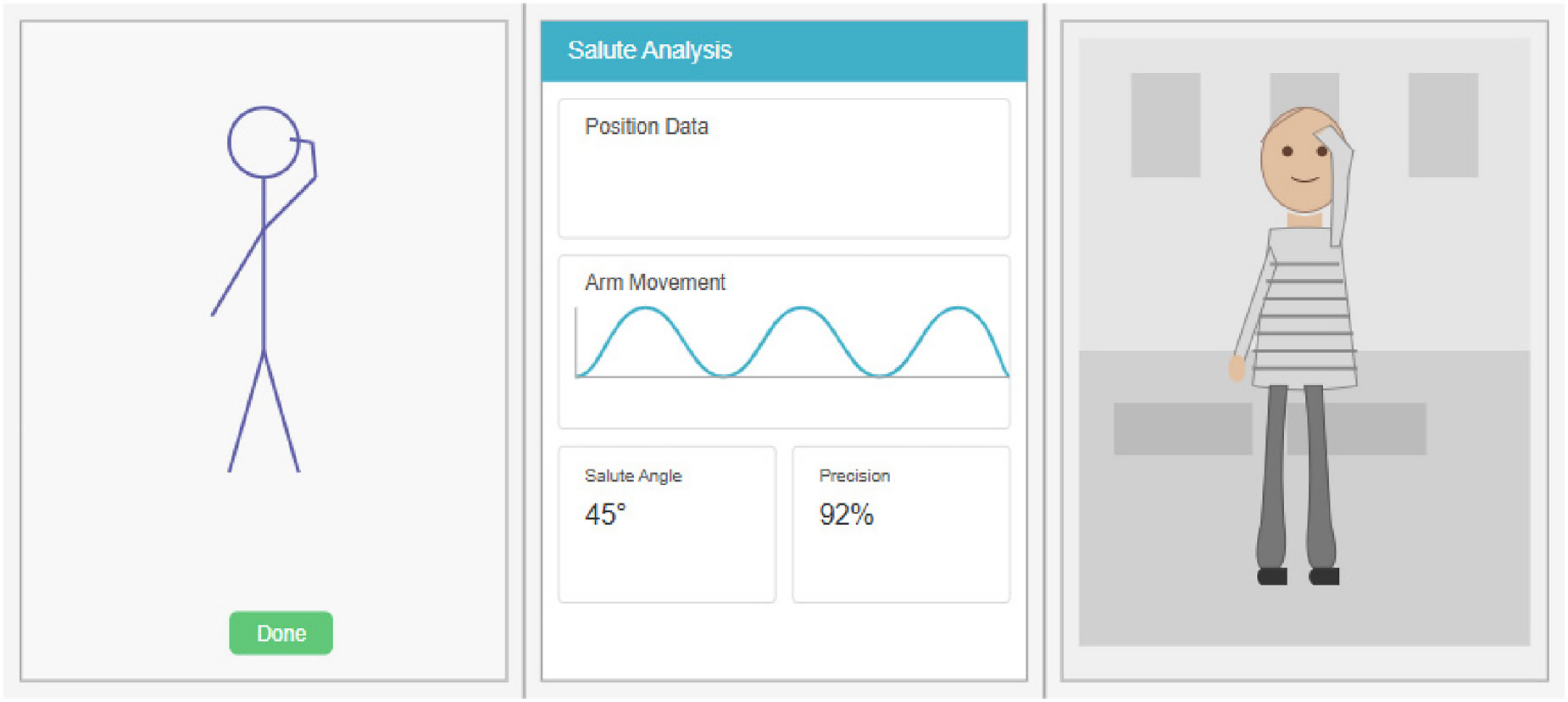
Analysis of the Fugel–Mayer hand salute test.

Motion analysis has come a long way, especially when it comes to helping people recover from strokes using video-based techniques that don’t require any contact or markers.^15^ The implementation of these techniques removes the requirement for bulky wearable sensors or markers, thereby offering a more intuitive and user-friendly approach to movement assessment. An analysis application was developed by extracting measurements from tracked body skeleton recordings as shown in Figure 1. This approach offers significant advantages for stroke survivors, who often experience restricted mobility and discomfort with conventional systems.^16^ The primary benefit of markerless systems lies in their capability to capture movements in real-time through the utilization of depth cameras. These cameras are typically more cost-effective and user-friendly when compared to traditional marker-based motion capture configurations. Using sophisticated CV algorithms for pose estimation and tracking, these systems can precisely analyze joint movements, trajectories, and various kinematic parameters. This offers a significant understanding of the functional capabilities of the upper extremities, eliminating the need for a controlled laboratory setting.

The noninvasive characteristics of these methodologies contribute to enhanced patient comfort during evaluations, leading to more authentic and natural movements.^17,18^ Furthermore, markerless techniques exhibit enhanced adaptability across diverse environments, such as clinics, rehabilitation facilities, and home settings. The adaptability of these systems significantly improves their application in remote monitoring and telerehabilitation, areas that are gaining prominence in contemporary healthcare practices. Markerless systems work with ML models to automatically look for patterns in motion data, classify functional impairments, and guess how well someone will recover. The implementation of these capabilities minimizes dependence on manual evaluation processes, thereby enhancing both the objectivity and efficiency of assessments.

### Objectives and scope of the review

This review aims to thoroughly examine the progress made in video-based, noncontact, markerless methodologies for evaluating the functionality of upper extremities in individuals who have experienced a stroke. The rising integration of CV and ML technologies in healthcare necessitates a thorough evaluation of their application in enhancing assessment accuracy, minimizing invasiveness and improving accessibility for clinicians and patients alike. This review systematically examines the methodologies, tools and applications utilized in recent studies to address existing gaps in the field. The review specifically examines critical components that determine the efficacy of markerless techniques. This analysis explores the various types and configurations of cameras employed, including monocular, stereo, and depth cameras, along with their associated hardware requirements. This analysis encompasses a thorough examination of pose estimation methods and algorithms, spanning from conventional techniques to advanced ML frameworks.

This review aims to identify the limitations and challenges present in current methodologies while also emphasizing potential avenues for future research. This review provides a comprehensive and organized overview of recent developments, intended to be a useful reference for researchers, clinicians, and technology developers focused on enhancing poststroke functional assessment methodologies. This comprehensive review aims to summarize advancements in markerless motion-capture systems for upper-limb functional assessment in stroke rehabilitation and to identify current challenges and future research directions.

## Methodology

This review uses a methodical approach to find, analyze, and combine existing research on assessing the functional capacity of the upper limb after a stroke using video-based, noncontact, markerless methods. The procedure is segmented into multiple essential phases to guarantee a thorough and impartial evaluation. A comprehensive literature search was performed utilizing various databases, such as PubMed, IEEE Xplore, Scopus, and Web of Science. The search covered studies published between 2012 and 2025. The retrieval of relevant articles was conducted using specific keywords and phrases, including “post-stroke rehabilitation,” “upper extremity functional assessment,” “markerless motion capture,” “non-contact video-based analysis,” “pose estimation,” and “machine learning in motion analysis.” Boolean operators (AND, OR) were utilized to enhance the search parameters and incorporate studies from various fields, including CV, biomechanics, and rehabilitation sciences.

### Inclusion and exclusion criteria

To ensure alignment with the aim of this review evaluating the use of video based, markerless systems for upper extremity assessment in poststroke rehabilitation—the following criteria were applied.

- The study was published in a peer-reviewed journal.
- The research involved human participants with poststroke upper limb impairment.
- The study employed video-based, markerless motion capture systems (e.g., RGB, depth, stereo cameras).
- The markerless system was used for the functional assessment of the upper extremity (e.g., joint kinematics, motion smoothness, ROM).
- The methodology involved noncontact techniques, excluding wearables or physical markers.
- Articles were written in English and provided full-text access.

Exclusion criteria were implemented to eliminate:

- Studies that did not involve stroke patients or did not assess upper limb function.
- Use of marker-based, wearable sensor-based, or invasive data collection methods (e.g., IMUs, EMG, gloves).
- Articles that lacked technical or clinical detail, or were not focused on functional evaluation (e.g., used markerless systems only for gesture recognition without clinical interpretation).
- Review articles, conference abstracts, editorials, or opinion pieces.

### Data extraction

The data extraction process concentrated on multiple essential elements to thoroughly assess the chosen studies. Data were collected regarding the type and number of cameras utilized, specifying the imaging hardware involved, such as monocular, stereo, or depth cameras, which are essential for the precise capture of motion data. The analyzed gestures were identified, encompassing a range of movements or activities, including reaching, grasping, and pointing, which are commonly assessed to evaluate upper extremity function. The studies documented the number of joints analyzed, detailing the specific upper limb joints included for functional assessment.

The review concentrated on the pose estimation methods utilized, emphasizing algorithms and frameworks including OpenPose, Mediapipe, and custom-developed models that facilitate precise motion tracking. Furthermore, analyzed the obtained parameters, which included quantitative metrics such as joint angles, trajectory smoothness, jerk, and ROM, providing a comprehensive understanding of motor function. The ML techniques utilized were evaluated, detailing the algorithms and models implemented for tasks including classification, prediction, and pattern recognition in motion data.

The accuracy and validation of these systems were evaluated, emphasizing performance metrics and reliability to determine their effectiveness in clinical applications. Lastly, the sample size and population characteristics were documented, including the number of participants and their demographics, to facilitate an understanding of the generalizability and scope of each study. The elements that were taken out provide a solid base for studying trends, limitations, and opportunities related to markerless, video-based methods for evaluating upper limb function after a stroke.

### Data analysis and synthesis

The analysis of the extracted data aimed to identify trends, strengths, and limitations present across the studies. Comparative analyses were performed to assess the methodologies, technologies, and outcomes documented. The assessment of practical utility in clinical and remote settings focused on parameters including system accuracy, feasibility, and applicability.

## Markerless motion capture

Markerless motion capture technology marks a transformative development in biomechanics, offering a noninvasive, straightforward approach to studying human movement. Unlike traditional systems that rely on complex configurations and physical markers, markerless solutions provide a natural alternative, particularly useful in clinical, sports, and rehabilitation contexts. Although early systems faced limitations such as reduced accuracy, sensitivity to environmental factors, and computational inefficiencies, these challenges have gradually been addressed.^19^

### Literature on markerless system

**Figure 2.**
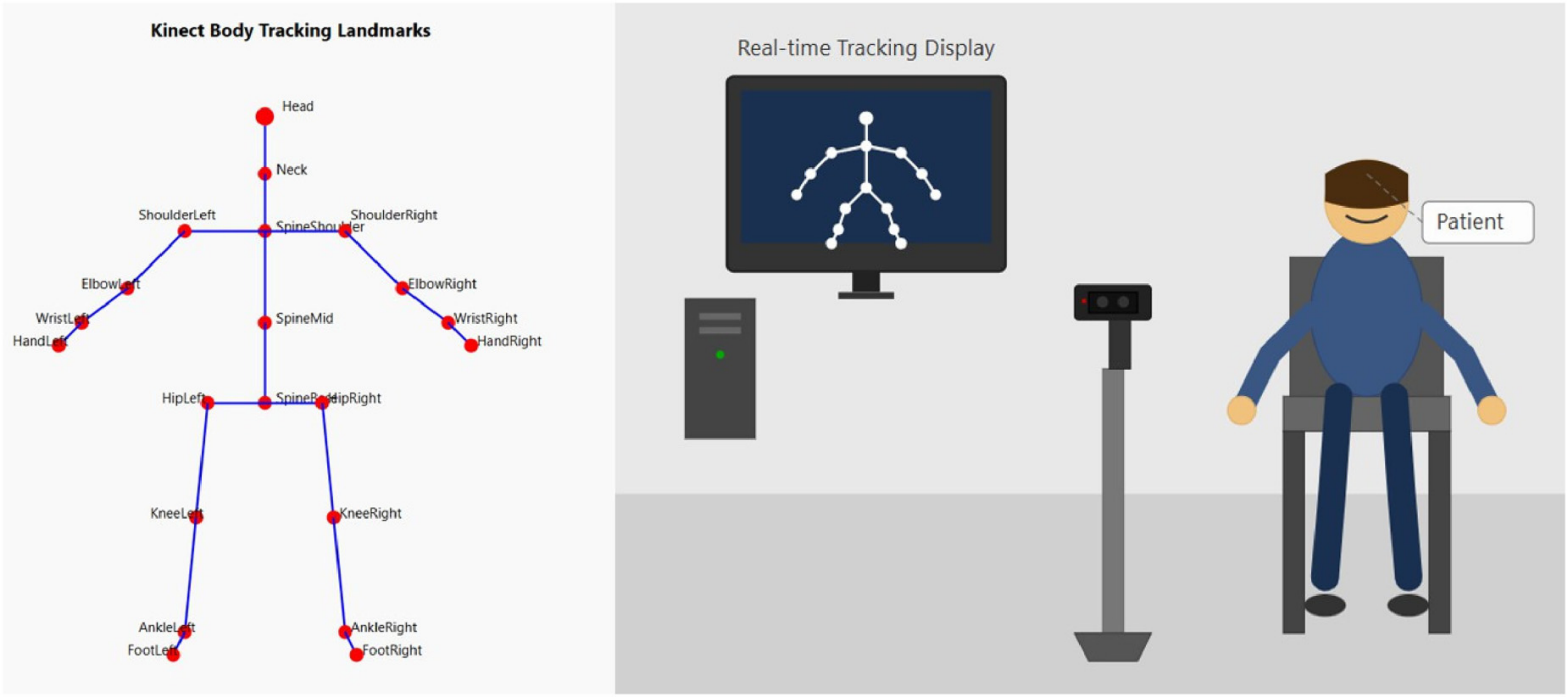
(a) 25-joint skeleton of KinectV2 and (b) the location of Kinect V2.^21^

**Figure 3. fig3-20552076251412636:**
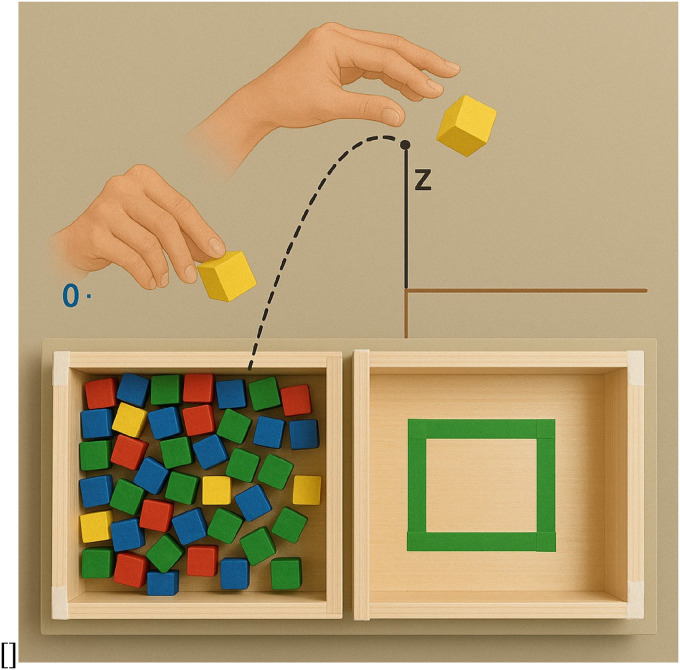
An overhead view of the modified Box and Block Test (BBT), with a green square highlighting the target area where participants aim to place the block. Markers on either side of the divider help define its plane and the cycles were identified by reorienting the coordinate system to better align with the task setup.^24^

The study developed and evaluated a fully automated markerless motion capture workflow using nine synchronized machine vision cameras (JAI sp500°c) capturing 1920 × 1080 resolution images at 200 Hz to analyze overground running, walking, and countermovement jumping in 15 participants, with full-body marker sets including upper and lower arms. The methodology employed deep learning-based 2D pose estimation using OpenPose's “body 25” model, followed by 3D fusion through occupancy maps, RANSAC-based outlier detection, bidirectional Kalman filtering for trajectory smoothing, and OpenSim inverse kinematics modeling to compute joint angles across the full kinematic chain including upper arm and forearm segments.^20^ The ML applications centered on deep convolutional neural networks for sparse keypoint detection in 2D images, with the full-body 6DoF model incorporating bilateral feet, shanks, thighs, pelvis, thorax, upper and lower arms, and hands, along with rigid clusters placed bilaterally on the upper arm and forearms. While the published results primarily reported lower limb accuracy (mean differences ranging from −0.1° to 10.5° for hip rotations and 0.7° to 3.9° for knee and ankle rotations), the comprehensive upper limb data collection demonstrates the system's capability for full-body biomechanical analysis. Strengths included the open-source, modular workflow enabling democratization of markerless technology and capability for comprehensive movement analysis, though significant limitations encompassed discrepancies in joint rotations (particularly up to 10° for hip internal/external rotation), error propagation through kinematic chains affecting measurement accuracy, reliance on inverse kinematics for certain rotations due to missing off-axis anatomical points and the requirement for multiple synchronized cameras which increases system complexity and cost. Issues like these discrepancies in joint rotations and reliance on multiple cameras remain areas for refinement in markerless motion capture technology. Despite these limitations, this validated full-body markerless approach, particularly its demonstrated upper limb tracking capabilities, provides a relevant foundation for upper limb assessment in rehabilitation contexts, offering a cost-effective alternative to traditional marker-based systems for clinical movement analysis and patient monitoring applications. Similarly, another study utilized a single Microsoft Kinect V2 motion-sensing camera, which captured XYZ coordinate data of 25 anatomical landmarks at 30 Hz sampling frequency without requiring physical markers. Its sensor is typically positioned strategically within the environment to ensure accurate data collection which is shown in Figure 2. The system analyzed four specific Wolf Motor Function Test-Functional Ability Scale (WMFT-FAS) tasks including forearm to table (side), forearm to box (side), extend elbow (side), and extend elbow with weight (side), utilizing the Kinect V2's built-in ML algorithms for 2D pose estimation with depth information. The methodology extracted 32 kinematic features categorized into endpoint kinematics (11 features such as maximum velocity, curvature index, interjoint coordination index), angular kinematics (18 features including maximum angular velocity and normalized mean absolute jerk), and other kinematics (three features such as maximum trunk displacement and path length). A feed-forward neural network served as the core assessment model, achieving promising performance with overall accuracy ranging from 0.87–0.96, F1-scores of 0.83–0.93, specificity of 0.94–0.98, sensitivity of 0.87–0.95, and AUC values of 0.93–1.00 when compared against 10 other ML algorithms including support vector machines (SVM), decision trees, and ensemble methods.^21^ Despite being cost-effective and portable, limitations included a small sample size (16 stroke patients), imbalanced clinical score distribution, and the exclusion of hand-specific tasks due to the Kinect V2's inability to track fine motor movements precisely, restricting the assessment to only four tasks rather than the complete 15-task WMFT-FAS evaluation. This study is highly relevant to upper-limb assessment for rehabilitation research as it demonstrates a practical, automated approach for objective motor function evaluation in stroke patients, laying groundwork for further development of accessible stroke rehabilitation tools that can enhance accessibility and consistency in clinical rehabilitation assessments while reducing the burden on healthcare professionals[Fig fig3-20552076251412636].

Based on the research presented, this study employed a dual-camera validation approach using an Azure Kinect camera (Microsoft, USA) for single camera markerless motion capture (SCMoCap) compared against a gold-standard 10-camera OptiTrack Prime 13 W marker-based system as reference. The researchers analyzed comprehensive physiological and functional upper limb movements typical of clinical assessments, including cup drinking tasks, with movements repeated three times across frontal and sagittal planes. The pose estimation methodology utilized Azure Kinect body tracking for markerless joint center detection, with joint angles calculated through inverse kinematics in OpenSim using Hamner's biomechanical model, while marker-based trajectories were processed with 20 Hz lowpass Butterworth filtering. Key parameters investigated included elbow flexion angles during activities of daily living (ADLs), with accuracy metrics showing good agreement (intraclass correlation [ICC] = 0.91°) but notable limitations including systematic underestimation at movement extremes (mean difference = −7.49°) and high variability (Root Mean Square Error [RMSE] = 16.30°).^22^ The study's strengths lie in its potential to facilitate at-home rehabilitation monitoring and enable more frequent data collection in clinical settings, particularly valuable for pediatric populations. While the system is affordable and user-friendly, it underperformed at motion extremes and was limited to a single task, suggesting room for enhancement. Additional limitations include sudden position changes at motion extremes and reduced accuracy at joint angle peaks and valleys. The study developed a smartphone-based system for wrist ROM assessment, achieving high interobserver reliability (ICC = 0.99). Instead of ML, they used traditional image processing techniques with Python and OpenCV. The process involved automatic hand and arm extraction, binarization using Otsu's method, GrabCut refinement and noise removal via connected-component labeling.^23^ While accurate and cost-effective, the system is limited to wrist flexion/extension and is sensitive to image quality. The study demonstrated that reliable ROM estimation can be achieved using image-based methods without ML, highlighting the potential for low-cost, accessible assessment tools in stroke rehabilitation scenarios.

The study evaluated the Theia3D markerless motion capture system in pediatric populations during the Box and Blocks Test (BBT) to analyze upper limb kinematics which is shown in Figure 3. Theia3D employs deep learning to estimate human poses from multiple camera views without physical markers. The study captured joint angles including shoulder flexion, abduction, internal/external rotation, elbow flexion, and forearm pronation. Root Mean Square Deviation (RMSD) values were under 6° for most joints, showing strong agreement with marker-based systems.^24^ Despite its overall accuracy, the system exhibited measurement biases for shoulder rotation and forearm pronation, particularly during rapid arm movements. The study confirmed Theia3D's potential for noninvasive, accurate upper limb motion analysis in pediatric clinical settings, while also noting the need for improvement in certain joint-specific estimations.

**Figure 4.**
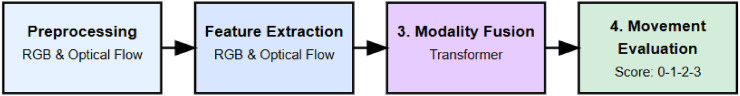
Architecture proposed by Kim et al.^25^

The research proposed a mobile solution for self-administered stroke rehabilitation using reach-to-grasp videos with a deep learning model combining RGB and optical flow data. The study utilized smartphone cameras (iPhone 13 Pro) as the primary sensing modality, eliminating the need for specialized equipment such as depth sensors or IMU devices. The research analyzed reach-to-grasp tasks where patients performed standardized movements involving reaching for and grasping a plastic cone placed at varying distances and angles, with assessment based on the Reaching Performance Scale global score criteria evaluating trunk displacement, movement smoothness, shoulder/elbow movements, and prehension quality. The overall architecture of the proposed model featured a sophisticated deep learning framework combining ResNet3D-50 backbone for spatiotemporal feature extraction, Transformer encoders for semantic dependency capture and a novel cross-attention mechanism with MLPMixer for RGB and optical flow data fusion. Key parameters investigated included four-level motor function scoring (0–3 scale), correlation with Fugl–Meyer Motor Assessment (FMA) scores, and NIHSS measurements, with the ML application achieving 86.08% accuracy and *R* = 0.93 Pearson correlation when compared against state-of-the-art action recognition methods.^25^ While the system demonstrated strong performance and offered 57 personalized exercises across four categories (postural/balance, ROM, strengthening, and task-oriented training), its lack of real-time feedback and limited task variety restricted its functionality. The system's strengths include accessibility through common smartphone technology, automated exercise recommendation based on motor function levels and integration with a comprehensive mobile application, while limitations encompass the absence of real-time performance feedback, focus on identical tasks creating classification challenges between high-performing patients (scores 2–3), lack of long-term effectiveness validation, and restricted task diversity. This research directly relates to upper limb assessment for rehabilitation by providing an objective, automated, and accessible platform for continuous motor function monitoring in stroke patients, enabling personalized exercise prescription and progress tracking in home-based rehabilitation settings, thus addressing the critical gap between clinical assessment and self-administered therapeutic interventions. The complete architecture is shown in Figure 4.

**Figure 5.**
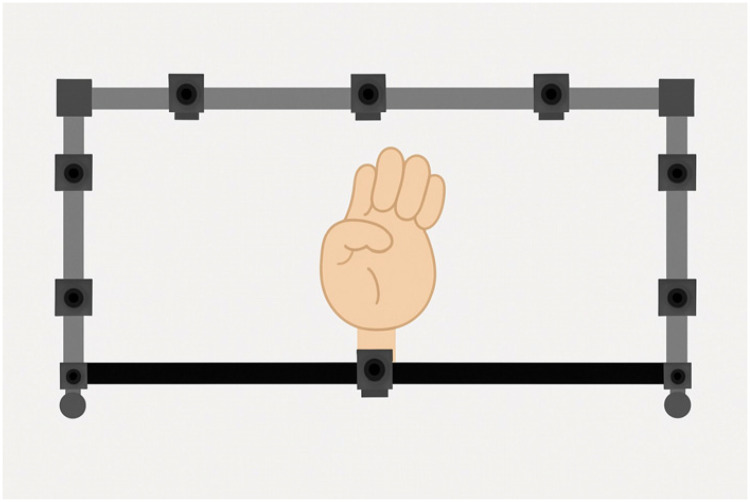
Eight camera configuration.^26^

The study created a novel markerless motion capture system for biomechanical arm and hand tracking using eight synchronized FLIR BlackFly S GigE RGB cameras (2048 × 1536 pixels, 30fps) to analyze American Sign Language gestures (“A,” “B,” “D,” “F,” “L,” “O”) performed across different hand orientations (supinated to pronated positions) the complex camera setups shown in Figure 5. The study employed multiple human pose estimation (HPE) algorithms including RTMPose, MeTRAbs-ACAE, MMPose, HRNetV2 and Freihand, comparing traditional two-stage reconstruction (triangulation followed by inverse kinematics) against an innovative end-to-end optimization approach. Their methodology integrated a comprehensive 28-degree-of-freedom biomechanical model (21 DoF hand + 7 DoF upper extremity) with differentiable biomechanics using MuJoCo-MJX for GPU acceleration, investigating parameters including joint angles, body scaling factors (humerus, ulna, radius, opisthenar area), and marker offsets through a multilayer perceptron that learned implicit trajectory representations. The ML application demonstrated superior performance with the end-to-end approach achieving 3.262 mm Mean Per Joint Position Error and 93.6% geometric consistency, representing a 39.4% improvement over traditional methods,^26^ showcasing strong spatial consistency but relied on controlled environments with optimal camera positioning and structured tasks. While the system's strengths include real-time processing capabilities, biomechanically constrained trajectories and elimination of physical markers, limitations include testing only able-bodied participants, restricted movement vocabulary, single-arm analysis, and dependence on laboratory-controlled settings that may not translate to clinical environments. This research directly supports upper limb rehabilitation assessment by providing precise, noninvasive biomechanical analysis capabilities that could enable continuous monitoring of patient progress, objective measurement of functional improvements and detailed kinematic assessment of complex hand-arm coordination during therapeutic interventions, making it highly relevant for developing automated rehabilitation evaluation systems.

The study developed a 3D markerless motion capture system using five synchronized GZ-RY980 cameras with 1 K (1920 × 1080 pixels at 120 Hz) and 4 K (3840 × 2160 pixels at 30 Hz) configurations to evaluate OpenPose-based pose estimation accuracy. The study analyzed walking, countermovement jumping, and ball throwing tasks representing 2D slow, 2D quick, and 3D quick movements respectively. Their methodology employed OpenPose (version 1.4.0), outputting 25 body keypoints, combined with Direct Linear Transformation for 3D coordinate conversion, investigating shoulder, elbow, wrist, hip, knee, and ankle joint positions using Mean Absolute Error (MAE) metrics. The deep learning application achieved 80% of measurements with MAE *<*30 mm and 47% *<* 20 mm, demonstrating markerless measurement capabilities without environmental constraints for sporting and clinical applications.^27^ Limitations included OpenPose tracking failures in 10% of cases (*>*40 mm error), requiring correction algorithms for incorrect joint position estimates and dependency on multiple cameras for optimal accuracy. The technology enables noninvasive tracking of upper limb movements during functional tasks, offering potential for clinical monitoring of patient progress without physical marker restrictions, particularly valuable for capturing natural movement patterns during therapeutic interventions. The study analyzed Microsoft Kinect V2 (optical depth-sensing at 30fps, 512 × 426 pixels, 25 joints) and Captiv L7000 (15 wireless inertial sensors with accelerometers/gyroscopes at 32–128 Hz). The study analyzed shoulder abduction/flexion and elbow flexion through static postures and dynamic movements with/without box handling to assess occlusion effects. Methodology employed iPi Soft for Kinect data integration and proprietary algorithms for Captiv's quaternion-to-joint angle conversion, investigating MAE, RMSE, and correlation coefficients against goniometer/angle scale standards. Automated pose estimation algorithms enabled joint detection and angle calculation in both systems. Kinect V2 performed well in nonoccluded scenarios while Captiv excelled under occlusion but incurred higher joint angle errors, underscoring the importance of application-specific system selection.^28^

The study developed a modular system combining binocular stereo vision and CNNs for 3D pose estimation, outperforming Kinect V2 in accuracy measures such as RMSE and MAE. However, sensitivity to environmental factors and lack of real-time processing remain hurdles. The system employed dual MV-CE060-10UC industrial cameras (480 mm baseline) analyzing standing postures with upper/lower limb movements across 25 body joint points. Their methodology integrated OpenPose algorithm with multilayer CNNs, using triangle similarity principles and Zhang's calibration algorithm for 3D coordinate reconstruction from binocular disparity. At 4450–4700 mm working distances, optimal performance achieved RMSE of 20.469, MAE of 16.408, and MAPE of 4.508, demonstrating superior joint length measurement accuracy compared to Kinect V2.^29^ Limitations included poor performance under strong lighting, nonreal-time processing, and increased right upper arm estimation errors due to environmental factors. The system targets stroke patient rehabilitation and community assessment, providing objective alternatives to clinical scales such as FMA and WMFT, enabling accurate, noninvasive measurement of arm joint lengths, and movements essential for monitoring stroke recovery and motor function improvement in rehabilitation settings.

The study integrated Kinect V2 depth imaging with wrist-worn IMUs for personalized upper-body motion capture. Their methodology employed personalized SMPL model with Levenberg–Marquardt optimization, integrating pose estimation with correspondence optimization through multiterm energy functions, achieving 40 DOF upper-body tracking. The statistical modeling approach utilized model personalization and IMU fusion for occlusion recovery, demonstrating 25.9% reduction in joint position errors compared to Kinect SDK (43.7% during occlusions), enabling detection of scapular elevation critical for stroke rehabilitation.^30^ Limitations included nonreal-time processing (1.5 s/frame), self-penetration artifacts, and frontal-facing restrictions. The system targets home-based upper-limb rehabilitation assessment, providing objective stroke patient monitoring without requiring multiple markers. The study compared Kinect V2 and Vicon for extracting kinematic synergies, reporting consistent cosine similarity values equal to or exceeding 0.60. Though promising for rehabilitation, the study faced challenges such as small sample size and motion occlusions. The study analyzed point-to-point reaching movements toward 9 targets across 3 orientations in 15 participants using Mixed-Matrix Factorization algorithm for statistical modeling of 10 rotational degrees of freedom, achieving reconstruction *R*^2^ = 0.94 for both systems.^31^ Results demonstrated markerless systems effectively capture motor control primitives comparable to gold standard marker-based systems, with intracluster similarity of 0.75–0.77. Limitations included poor detection of fine movements, distal joint tracking issues, and motion occlusions. Applications target clinical motor control assessment, neurological rehabilitation monitoring, and home-based telerehabilitation without expensive marker-based setups. The study developed a portable markerless hand motion capture system using Leap Motion Controller (LMC) with three infrared cameras, offering a revised taxonomy of grasps for ADLs. Accessibility was a strength, but accuracy and participant diversity need refinement. The study analyzed daily activities across 13 participants over 62 h using LMC-based pose estimation capturing 22-point hand model at 120 Hz with k-means++ clustering algorithm. Results achieved 9.89 mm accuracy compared to VICON standard, with R-squared values of 97.74% and 96.98% for left/right hands, identifying 38 left-hand and 22 right-hand grasp types.^32^ Strengths included portability, cost-effectiveness (£521.61), markerless operation, and automatic analysis. Limitations encompassed lower accuracy than laboratory systems, occlusion issues, lighting sensitivity, and limited participant diversity. Applications target upper-limb prostheses development and updated grasp taxonomies for modern daily activities.

Here's the corrected version with the citations moved to the proper locations and the first sentences fixed: The study assessed a multi-kinect system for estimating joint reaction forces in exoskeleton users. It utilized multiple Microsoft Kinect V2 cameras with RGB and Time-of-Flight sensors for exoskeleton-assisted gait analysis, tracking upper limb biomechanics during walking trials with instrumented crutches. Pose estimation employs Kalman filtering for multisensor fusion, converting 25-joint skeletons to virtual markers for OpenSim biomechanical modeling. Key parameters include RMSE validation, force normalization by body weight, and gait phase analysis across 12 degrees of freedom (bilateral shoulder/elbow forces in three directions). Machine learning applications assessed a multi-kinect system for estimating joint reaction forces in exoskeleton users, achieving RMS errors under 2% of body weight compared to gold-standard marker-based systems,^33^ offering strengths of low cost (3000), real-time processing, and contact-free operation for rehabilitation monitoring, but with limitations including 1-s initialization delays, force platform dependency, reliance on stationary setups, and validation limited to a single participant rather than diverse patient populations. The study validated the Microsoft Kinect v2 (RGB-depth-infrared sensor, 30 Hz) against the gold-standard Vicon marker-based system (100 Hz) for upper limb kinematics during seated reaching tasks. Twenty-six healthy participants performed horizontal reaches while holding dumbbells to simulate stroke-like movements, with data processed using 2.5 Hz low-pass filtering.^34^ The study investigated 17 kinematic parameters including joint angles, movement efficiency, planning, smoothness, and speed metrics, plus the Proximal Arm Non-Use score. Results showed excellent reliability (ICC > 0.90) for trunk compensations and displacements, moderate reliability (ICC 0.50–0.88) for movement time and mean velocity, but poor reliability (ICC < 0.50) for instantaneous measures such as peak velocity due to noise and elbow occlusion. It's effective for gross motion analysis but moderately reliable for finer movements like elbow extension, confirming its potential as an affordable rehabilitation tool. The Kinect's strengths include markerless operation and clinical feasibility for stroke rehabilitation monitoring, though limitations include poor elbow tracking and inadequate precision for fine kinematic analysis, making it suitable for global motion assessment but requiring caution for detailed joint measurements.

The study included nine stroke survivors and 10 healthy adults who performed specific motions using a tabletop rehabilitation robot. The healthy participants performing reach-forward-back and reach-side-to-side motions, with healthy participants simulating three compensatory strategies (shoulder elevation, trunk rotation, lean-forward). By analyzing 3D trajectories of upper-body joint positions, researchers aimed to classify postures using ML models.^35^ The SVM classifier accurately detected lean-forward compensation in healthy participants (AUC: 0.98, F1-score: 0.82) and showed moderate success for trunk rotation (AUC: 0.77, F1-score: 0.57), but struggled with shoulder elevation (AUC: 0.66, F1-score: 0.07). The RNN classifier, which considers the timing of movements, showed similar results. For stroke survivors, however, both classifiers struggled, with significantly lower accuracy across all compensations. Strengths include marker-free operation and real-time potential, while limitations encompass inadequate detection in real patients due to subtle compensations and camera occlusion. To address these challenges, future efforts should focus on refining the setup, including adjusting motion ranges, camera angles, seat height, and exercise intensity and recording full-therapy sessions to capture more meaningful data.

This study validated the Microsoft Kinect v2 depth camera against a 6-camera 3D motion analysis system (OptiTrack Prime 41) and goniometry for measuring shoulder kinematics, highlighting Kinect v2's potential as a reliable and cost-effective tool for assessing shoulder ROM and arm motion smoothness while overcoming limitations of traditional methods. Involving 10 male participants, the research evaluated static shoulder ROM measurements (flexion, abduction, external/internal rotation) and dynamic point-to-point arm movements to analyze motion smoothness during dynamic movements. Pose estimation used Kinect v2's skeletal tracking algorithms providing 3D coordinates of 25 joints, compared against marker-based tracking for the gold-standard system. Parameters investigated included shoulder ROM angles and motion smoothness metrics (peak-to-mean velocity ratio, acceleration-to-movement time ratio, number of velocity peaks), analyzed using Pearson correlation and Bland–Altman analysis. Results showed that Kinect v2 closely matched the accuracy of 3D motion analysis, with a margin of error under ±8° for ROM measurements—significantly better than goniometry's ±10° and effectively assessed motion quality with strong correlations with 3D analysis data.^36^ Strengths include marker-free operation as a practical, user-friendly alternative for clinical evaluations, eliminating the need for bulky, expensive equipment while maintaining precision, while limitations encompass small sample size and comfort-based measurements, indicating clinical potential for comprehensive shoulder assessment.

The study presented a markerless motion capture system utilizing stereo action cameras for assessing fine hand motor skills via color-based object tracking. Through three validation experiments, the system demonstrated high accuracy with distance estimation errors below 5 mm and angular errors of 0.02–0.09 radians, successfully identifying motor performance improvements in chopstick manipulation tasks.^37^ While offering cost-effectiveness and accessibility compared to traditional marker-based systems, limitations include nonreal-time processing capabilities, limited sample size, lighting sensitivity, high computational requirements, and inability to capture compensatory movement strategies, though it shows promise for rehabilitation research and clinical motor assessment applications.

The study examined how well the LMC, a markerless infrared-based system, measures upper limb movements compared to the gold-standard Optotrak system. Involving 44 participants across three experiments (visually guided reaching, Fitts-type aiming tasks, and reach-grasp-place sequences), it looked at movement time, deceleration intervals, peak velocity, and spatial accuracy. The LMC proved to be an affordable and portable tool, but it underestimated movement duration and peak velocity, with average errors of 40 ± 44 ms and 0.024 ± 0.103 m/s and showed issues such as inconsistent sampling rates, high data loss (30% vs. 5% for Optotrak), and less reliable spatial accuracy.^38^ Despite these drawbacks, the LMC's accessibility makes it a promising option for clinical assessments, rehabilitation, and motor research, particularly in settings where cost and portability are priorities over precision, though not recommended for short-duration movements or studies requiring detection of small temporal differences (*<*100 ms).

Highlighting developments in markerless motion capture systems, the survey assesses their potential for clinical, sports, and rehabilitation applications. The accuracy, dependability, and usability of several systems—including Kinect V2, Azure Kinect, OpenPose, and Leap Motion—were evaluated across a range of workloads and demographics. Although markerless systems provide accessible and affordable substitutes for conventional marker-based configurations, issues including task-specific limitations, restricted real-time processing, and environmental sensitivity still exist. The results highlight how feasible these technologies are becoming for practical uses, with room for improvement in terms of precision, flexibility, and validation for larger populations. The summary of this survey is provided in Table 1.

**Table 1.** Comparison of markerless motion capture systems across applications, strengths, and limitations.

Reference	System used	ML method	Pose estimation	Gesture/task	Population	Parameter assessed	Strengths	Limitations		Applications
^20^	9-camera (JAI sp5000c)	Deep learning	OpenPose Body-25	Walking, running, jumping	General movement	Upper arm + forearm	End-to-end automation	Limited task diversity		Rehab, sports
^21^	Kinect V2 (1 cam)	FFNN	Kinect pose, Body-25	WMFT (4 tasks)	16 stroke, 10 healthy	Angle, velocity, smoothness	Low cost, high accuracy	Basic movements only		Stroke rehab
^22^	Azure Kinect	—	Azure body tracking	Drinking task	Pediatrics	Elbow flexion (ICC = 0.91)	Strong agreement	Poor extremes, RMSE = 16.3°		Clinical/home rehab
^23^	Smartphone ROM	Image processing	Traditional CV	Wrist flex/ext	33 adults	ROM	Highly accessible	Depends on image quality		Telehealth
^24^	8 Theia cameras	Deep learning	OpenPose Body-25	Box & Block	21 participants	Shoulder, elbow, wrist	Advanced framework	Small sample		Sports science
^25^	iPhone 13 Pro	Deep learning	Task-specific	Reach-to-grasp	100 stroke survivors	Qualitative motion	6.08% clinical-grade	No real-time feedback		Rehab
^26^	8 FLIR Black-Fly S	Deep learning	Hand5, COCO WB	6 gestures	13 participants	MCP, pro/sup angles	High binocular accuracy	Controlled environment		Clinical tracking
^27^	5 GZ-RY980 cams	Deep learning	OpenPose Body-25	Walking, jumping, throwing	2 adults	Limb angles	80% ¡ 30 mm error	10% tracking failures		Accuracy eval
^28^	2 Kinect + captive sensor	—	Kinect pose	Static + dynamic	12 exoskeleton users	ROM	Good estimation	Occlusion issues		Rehab assessment
^29^	Binocular vision	CNN	OpenPose Body-25	5 standing actions	—	Arm/forearm length	Outperforms Kinect	Not real time		Clinical rehab
^30^	Kinect V2	Stats + optimization	SMPL model	Multiple tasks	6 adults	Joint position	25.9% improvement	Slow		Telerehab
^31^	Kinect V2	MMF modeling	Kinect pose	Reaching	15 adults	ROM	Synergy validation	Hand discrepancies		Clinical assessment
^32^	Leap Motion	Unsupervised	LMC pose	Daily activities	13 adults	Finger angles	Portable, cheap	Occlusion, 9.89 mm error		Rehab, therapy
^33^	4 Kinect setup	Kalman filter	Kinect pose	Walking	1 exo user	Joint angles	Force accuracy	Needs platform	force	Force analysis
^34^	Kinect V2 vs. Vicon	Stats	Kinect pose	Dumbbell reach	26 adults	ROM	Reliable trunk comp.	Poor tracking	elbow	Stroke rehab
^35^	Kinect V2	SVM/RNN/LSTM	Kinect pose	Reach tasks	9 stroke, 10 healthy	Postural class	Multiclass ability	Class imbalance		Rehab assessment
^36^	Kinect V2	Stats	Kinect pose	Reaching	10 adults	Shoulder angle	Better than goniometry	Small sample		Clinical assessment
^37^	GoPro Hero 9	CV	Object tracking	Chopstick test	11 adults	Motor performance	¡5 mm accuracy	No joint tracking		Research
^38^	LMC + Opto-trak	None	LMC pose	Reaching	44 adults	Velocity, smoothness	Portable	30% data loss		Clinical assessment

FFNN: feed-forward neural network; RMSE, root mean square error.

### Critical appraisal of markerless systems

While Table 1 provides a detailed comparison of markerless motion capture systems, a critical appraisal reveals varying levels of methodological robustness and clinical readiness. Systems such as Theia3D and Azure Kinect exhibit strong accuracy in joint angle estimation (often RMSD *<* 6°), supporting their potential clinical utility. However, many studies are constrained by small sample sizes, often lacking representation from actual stroke survivors. Instead, healthy subjects simulating impairments are frequently used, reducing ecological validity.

A recurring limitation is the dependency on controlled lab environments; many systems underperform in natural or home-based settings due to occlusion, lighting changes, and camera misalignment. Furthermore, few studies explore long-term applicability or real-time responsiveness—key features for telerehabilitation and patient feedback. Although noninvasiveness and affordability are major strengths, inconsistent validation methods (e.g., lack of comparison with gold-standard systems), and limited task generalizability remain critical barriers to adoption. Thus, while the systems reviewed show significant promise, more comprehensive validation in clinical contexts is essential.

### Literature on advanced markerless motion capture

This study utilized a Microsoft Kinect depth-sensing camera operating at 30 Hz to capture three-dimensional joint movements for automated FMA scoring in hemiplegic stroke patients, achieving a strong correlation of 0.873 with therapist-assessed scores and providing motion smoothness analysis metrics. The system tracked 31 variables across head, shoulder, elbow, wrist, and hand joints while analyzing 13 upper extremity movements including flexor/extensor synergy patterns and volitional movements from the 33-item FMA protocol.^39^ The methodology employed real-time joint tracking with data normalization, investigating joint angles, interjoint distances, and movement smoothness via integrated squared jerk analysis correlated with Brunnstrom recovery stages. An artificial neural network with principal component analysis (PCA) achieved 65–87% prediction accuracies per item using 8–10 fold cross-validation on 82 motion captures. While offering cost-effectiveness, accessibility, and quantitative assessment for potential home-based telerehabilitation, the system faced limitations including occlusion challenges, inability to track fine hand movements, restriction to 13 of 33 FMA items, data imbalance issues, and homogeneous participant groups requiring larger datasets for improved clinical accuracy. Complementing this approach, a study assessed MediaPipe Pose (MPP) for real-time accuracy and HybrIK for enhanced depth estimation in 3D HPE. The study utilized monocular RGB cameras (1280 × 720, 30 FPS) analyzing yoga poses, cycling, pitching, rowing, and walking motions through two methodologies: MPP with 33 landmarks using Blaze Pose Model and HybrIK with 29 landmarks using inverse kinematics and SMPL model.^40^ Parameters investigated included 3D joint coordinates, joint angles via 26-DoF humanoid model, link lengths, motion smoothness, and outlier detection for left/right inversions under varying lighting and occlusions. Machine learning applications employed uDEAS optimization achieving cost function improvements from 0.0147 to 0.0046 (69% reduction), with MPP demonstrating superior real-time performance and facial landmark recognition but depth estimation limitations, while HybrIK showed better depth estimation but reduced accuracy in complex joints and object differentiation. The suggested framework provides structured correction for joint misdetections and left/right inversions using link length derivatives, mean interpolation, and median filtering, improving accuracy in challenging conditions. Both approaches faced limitations including occlusion handling, lighting sensitivity, algorithm-specific issues, and real-world environmental constraints, with applications extending to sports analysis, healthcare rehabilitation, fitness monitoring, surveillance, and cost-effective motion capture alternatives. A study evaluated the efficacy of integrating an RGB-D camera (positioned 1.5 m high, 3 m from subjects) with AI-driven convolutional neural networks to quantify shoulder ROM across seven tasks: abduction, adduction, flexion, extension, external rotation (ER1), external rotation in 90° abduction (ER2), and internal rotation in 90° abduction (IR2). The pose estimation methodology employed stacked hourglass networks detecting 2D skeletal structures of upper limb joints (shoulder, elbow, wrist) and trunk axis, with AI algorithms automatically superimposing 3D skeletons onto subjects for tracking and measurement in predefined planes.^41^ Parameters investigated included intertester ICC, concordance correlation coefficients (CCC), mean bias, and limits of agreement between visual estimation (EyeREF), goniometric measurement (GonioREF), and automated software (shoulder ROM) methods. Machine learning applications achieved excellent accuracy with ICC values constantly excellent for vertical plane motions, very good for rotations, and overall good-to-excellent CCC between methods, with measurement biases remaining below 10° compared to conventional goniometry. The system demonstrated strengths in providing standardized, cost-effective, markerless shoulder RoM measurement while facing limitations including lowest accuracy for ER1 (CCC = 0.82), difficulty standardizing horizontal plane measurements, and need for validation in pathological populations, with applications extending to clinical assessment, consultation evaluations, paramedic data collection, and daily activity movement analysis.

This study presented OpenPoint, a webcam-based system that employs MediaPipe's deep learning library for real-time tracking of 21 hand landmarks to evaluate upper extremity proprioception via finger-to-finger pointing tasks under varying visual conditions (full, partial, blindfolded) and proprioceptive scenarios (real vs. fake hand targets). Using an HD 1080P webcam, the system measured frontal-plane pointing error normalized by hand size across young, older, and stroke participants, accurately detecting proprioceptive deficits in stroke patients and demonstrating significant correlation with independent robotic assessments (*r* = 0.617).^42^ This cost-effective solution is adaptable for individuals with severe motor impairments, providing potential applications in telemedicine and rehabilitation settings. The approach utilizes two-dimensional depth estimation through MediaPipe's CV framework and has been validated primarily in controlled laboratory conditions with specific populations, establishing a foundation for accessible quantitative proprioception assessment in clinical practice.

This study assesses egocentric video using the Hand Object Detector deep neural network as an innovative method for evaluating hand function in stroke survivors in 24 participants across laboratory and home settings, presenting the hand use ratio and hand role ratio as metrics for outcomes. This approach captures both static and dynamic hand movements, offering advantages over traditional accelerometer-based methods. The hand-use ratio demonstrates significant correlations with clinical assessments including the Motor Activity Log (*r* = 0.80 for amount of use, *r* = 0.79 for quality of movement), FMA-UE (*r* = 0.60), and ARAT (*r* = 0.44), confirming its applicability for evaluating hand performance in practical environments and demonstrating superior performance compared to previous arm use ratio studies.^43^ The hand-role ratio demonstrates a lack of significant correlation with clinical measures, likely due to limited bilateral task data and the complexity of manual annotation requirements, which restricts its practical application. However, the study's validation primarily in mild impairment cases and privacy considerations for home recording present limitations, while applications extend to objective rehabilitation outcome measurement beyond clinical settings. A system was introduced that recognizes human actions by combining movement tracking, object recognition, and feature modeling using Kinect depth sensors, dynamic instants methodology, and Latent Structural SVM. The framework connects human movement and object interactions to identify complex fine-grained cooking manipulations such as cutting, pouring, and sprinkling. Tested on a new Cooking Action Dataset and VISINT dataset, the model achieves 84% accuracy versus 75% for feature-based and 65% for pose-only approaches.^44^ By focusing on movements and object interactions, this approach enables applications in surveillance systems, assisted living monitoring, and smart home technologies answering queries like “Where did I put the knife?” The system demonstrates object tracking through manipulation sequences while overcoming traditional camera limitations through depth sensing technology. A system was introduced that uses computational intelligence to evaluate motor function in poststroke patients through the analysis of video data, utilizing HRNet for pose estimation and extracting 24-dimensional features (10 keypoint coordinates and 4 joint angles), employing deep learning models such as LSTM and TCN (with TCN demonstrating superior performance). According to NIHSS standards, the system has a high level of precision (92.1% for binary classification and 89% for three-class classification) because it uses skeletal key points to determine how bad motor weakness is.^45^ The system demonstrates scalability, cost-effectiveness, and noninvasiveness; however, a limited dataset (23 patients), environmental factors, and a focus on upper body movements constrain its applicability.

Framework validation utilized two classification approaches employing an ANN that outperformed five other ML models. The first approach concentrated on binary classification, distinguishing between control subjects and patients. The second approach centered on multiclass classification, categorizing patients into three severity classes (mild, moderate, severe) based on SDMT clinical assessments. Using 22 extracted kinematic parameters from 89 video recordings (31 patients, 58 controls) with PCA/LDA dimensionality reduction, technical validation revealed accuracies reaching 96.67% for binary classification and 92.22% for multiclass classification.^46^ This ANN-based approach effectively addressed both classification tasks, underscoring its practicality in clinical assessment scenarios.

The study explored why some stroke survivors with good arm motor function after rehabilitation still struggle to use their arms in daily life. This retrospective study analyzed data from 78 participants, dividing them into two groups: those with good motor ability but low daily arm use and those who didn’t fit this pattern. They identified five key factors from standardized clinical assessments: motor function (FMA-UE), quality of movement (MAL-QOM, WMFT-Quality), daily arm use (MAL-AOU), and self-confidence (Stroke Self-Efficacy Questionnaire). Using four ML algorithms, predictive models were developed with accuracies ranging from 75% to 94%, with k-nearest neighbors performing best.^47^ However, the study's small sample size and need for external validation limit generalizability. The findings suggest that focusing on motor skills, daily use of the arm, and self-efficacy during rehabilitation could help create more personalized programs to reduce arm nonuse and improve recovery outcomes for stroke survivors. The study evaluated six algorithms (Logistic Regression, Decision Tree, Random Forest (RF), Random Forest with Hyperparameters Tuning, SVM, and Deep Neural Network) to classify 429 stroke participants versus 465 controls using 12 kinematic parameters from an Arm Position Matching task with a Kinarm Exoskeleton robot. Using tenfold cross-validation, models achieved 82.4% accuracy with AUC values of 0.85–0.90, outperforming traditional cutoff techniques.^48^ Logistic Regression achieved the highest AUC (0.900), while RF showed superior recall and F1 scores. Variability parameters emerged as most important for classification. Random Forest's ensemble approach effectively integrated multiple kinematic features and identified complex parameter relationships, demonstrating its power for clinical ML applications.

This study explores a simple and affordable way to measure movement quality using Microsoft Kinect motion capture technology. People with chronic hemiparesis performed arm movements that were recorded and analyzed using PCA. The results showed strong correlation (*R*^2^ = 0.868) between automated scores and trained human raters, performing as well as an average of 3.4 human assessments.^49^ This approach provides an accessible and objective way to evaluate poststroke arm impairments, with potential applications in clinics, home-based therapy, and telerehabilitation, making reliable measurement tools more affordable and standardized across healthcare settings.

The study aimed to determine the main factors influencing recovery outcomes and examine whether ML could accurately forecast upper limb recovery (UL) in patients with subacute stroke, along with using explainable AI (XAI) methods to interpret ML model predictions and comparing ML performance against traditional statistical approaches for predicting individual recovery scores. To do this, the researchers analyzed the data and assessed the relevance of different variables using XAI approaches in combination with a RF model.^50^ The results showed that ML models outperformed traditional statistical methods. It's interesting to note that the most crucial characteristics were the same for all XAI techniques, indicating consistent and dependable results. The degree of baseline motor impairment as determined by basic clinical scales was, predictably, the most important factor affecting recovery. All things considered, the work demonstrates the ability of ML to not only produce precise predictions for stroke recovery but also to use XAI techniques to make those forecasts easier to comprehend. This can provide doctors with the resources they require to effectively plan and track recovery.

The study explores how affordable vision-based systems can help identify compensatory postures in stroke patients during robot-assisted upper-body rehab. Using a dual RGB camera system with OpenPose software, researchers worked with 10 stroke survivors performing scripted reaching tasks and tracked their upper body movements.^51^ They found common issues such as forward trunk leaning, trunk rotation, shoulder elevation and, less often, insufficient elbow extension. Two ML models were used to detect these postures with varying accuracy—performing best for elbow extension (F1 = 0.73–0.80) but poorly for shoulder elevation (F1 = 0.50–0.53). The results show promise for real-time feedback in rehab, helping patients and clinicians correct movements and improve recovery outcomes, though the study's small dataset size and technical limitations indicate need for further validation before clinical implementation.

A classifier based on SVM was presented to evaluate the recovery of finger movements in stroke patients, utilizing data obtained from the LMC with 24 stroke patients. Through the analysis of kinematic parameters such as peak angle and velocity, the system correlates results with Brunnstrom's recovery stages, resulting in an accuracy of 86.3% (95% CI: 0.857–0.869).^52^ Limitations identified in this study encompass a small sample size, challenges associated with sensors under specific conditions and an emphasis on finger flexion and extension movements. A method for gait analysis was presented that utilizes DL techniques with single-camera video input. It incorporates CNNs and OpenPose for the purpose of 2D pose estimation. The system was evaluated using a dataset comprising 1792 videos from 1026 children with cerebral palsy.^53^ The system accurately predicts gait parameters such as walking speed, cadence, knee flexion angles, and Gait Deviation Index and GMFCS scores, displaying correlations ranging from 0.73 to 0.83 when compared to motion capture data, approaching theoretical accuracy limits imposed by natural gait variability. The method demonstrates accessibility and scalability, making it appropriate for both clinical and home applications. However, the method has limitations including single sagittal-plane view restrictions and validation from only one clinical center.

A Microsoft Kinect v2 sensor was employed as a cost-effective alternative to traditional marker-based 3D motion capture systems for upper limb assessment. Four daily living tasks were analyzed (hand-to-shoulder, hand-to-mouth, combing hair, hand-to-back pocket) focusing on shoulder and elbow joint movements. A three-layer LSTM neural network refined Kinect's skeletal tracking data using leave-one-subject-out cross-validation, with performance evaluated via CMC, RMSE, ROM, and PTA parameters.^54^ The deep learning approach achieved CMCs *>*0.93 for flexion/extension movements and *>*0.8 for most joint angles, reducing mean deviations to under 5°. While offering portability and cost-effectiveness for clinical rehabilitation, limitations include handling occlusion scenarios and reduced accuracy in certain movement planes, making it suitable for small clinics and home-based monitoring despite not fully replacing gold-standard systems.

The Kinect's kinematic measurements are enhanced, resulting in high accuracy with CMC values exceeding 0.93 across multiple functional tasks, including hand-to-mouth actions and hair combing. However, a limited participant pool, task-specific performance and the lack of dynamic validation constrain the system's cost-effectiveness and practicality. The MPL-CNN model combines Mediapipe key point extraction with CNN and LSTM methods to test rehabilitation training for the upper limbs. Mediapipe identifies 33 body keypoints and 21 hand keypoints, creating 258 total features processed through 30-frame temporal sequences. The system demonstrates an accuracy of 97.54% in classifying movements according to Fugl–Meyer standards with real-time processing capabilities, providing a cost-effective and scalable solution for home-based rehabilitation using standard RGB cameras,^66^ though requiring controlled environments and tested on healthy participants rather than actual stroke patients.

This study validates two hand-tracking frameworks, Google MediaPipe Hand (GMH) and its enhanced version GMH-D, against OptiTrack motion capture for clinical assessment applications. Testing three Parkinson's disease evaluation tasks (hand opening-closing, single finger tapping, multiple finger tapping) across varying distances, viewing angles, and speeds, GMH-D demonstrated superior spatial accuracy with *<*15% error and *>*0.97 correlation, while GMH showed limitations in tracking finger contact phases and self-occlusions.^55^ Both frameworks achieved real-time performance (*>*30 fps) without GPU requirements. The findings highlight GMH-D's clinical potential and emphasize the importance of rigorous validation for deep learning–based frameworks before clinical deployment, while identifying areas for future research including lighting conditions and complex hand movements.

This study introduces an affordable upper limb rehabilitation system for stroke patients using deep learning. With a basic camera deployable on smartphones, it records patients performing elbow flexion exercises (80°–100° range) and uses Faster R-CNN and HRNet algorithms to analyze arm movements. An LSTM neural network with ProbSparse Self-Attention mechanism achieves 94.1% accuracy, clinically validated with 81 stroke patients using Brunnstrom staging criteria.^56^ Unlike expensive Kinect systems requiring controlled setups, this approach enables home-based rehabilitation through mobile devices, works in various environments and remains cost-effective. By providing precise movement analysis, it helps track progress and tailor treatments, making quality rehabilitation more accessible to patients.

Here's the corrected version with the citation moved to the proper location and the first sentence fixed: This study focuses on making stroke rehabilitation more accessible by using a smartphone app powered by MediaPipe to track hand movements. While healthcare professionals traditionally struggled with assessing complex hand motor functions and the 2019 Fahrenheit system required specialized infrared equipment, this research validated MediaPipe as a viable alternative. Testing 40 stroke patients across different severity levels, the study found moderate-to-high correlations between MediaPipe's smartphone-based tracking and Fahrenheit's specialized system for measuring peak angles and angular velocity.^57^ Despite some higher noise levels and challenges with severe cases, the validation confirmed smartphone-based assessment can match specialized equipment standards. This breakthrough enables easier, more accessible rehabilitation tools for both clinic and remote care. Future ML enhancements could further improve accuracy, making high-quality hand function assessment truly accessible to everyone who needs it.

DRome is an innovative mobile vision system utilizing Google's MoveNet architecture and TensorFlow for Android deployment, designed for real-time ROM evaluation in stroke and musculoskeletal disorder rehabilitation.^58^ The system achieves highly accurate joint angle measurements with RMSE of 3.70–7.29° across shoulder and elbow movements, significantly improving upon the base MoveNet model's 12–15° RMSE through optimized ML algorithms (polynomial regression, SVR, MLP). Validated against the gold standard Vicon motion capture system using 13 healthy participants, DRome demonstrates real-time processing at 11–16 fps with *<*25 MB memory usage while meeting clinical accuracy requirements (*<*5 *degree* error). However, limitations include reduced performance with darker skin tones, extensive tattoos, and dependency on controlled camera positioning. This cost-effective, accessible tool enables active patient participation in rehabilitation and has potential to revolutionize MSD management, particularly in medically underserved areas with limited healthcare access.

Using CV technology, the study investigates novel approaches to enhance hand rehabilitation for stroke patients. It presents a vision-based rehabilitation method that eliminates the need for frequent hospital stays by allowing patients to complete activities such as the BBT and the Sollerman Hand Function Test at home. The system employs advanced 3D hand landmark detection using MediaPipe framework, representing a significant improvement over previous 2D models, particularly in handling curved alphabets and thumb keypoint localization.^59^ This cost-effective approach requires only a standard RGB camera without specialized equipment or high computational resources, providing real-time feedback to patients during exercises. To assist patients in monitoring their development, the system records hand and finger movements and provides performance scores. Experiments conducted over four weeks with 15 participants revealed significant gains in hand functionality, underscoring the potential of CV to facilitate motor recovery and increase accessibility to rehabilitation. According to the results, incorporating this technology can improve patient outcomes and lessen the need for conventional clinical evaluations.

The study develops a novel algorithm using MediaPipe framework to estimate five upper limb movements (shoulder internal/external rotation, elbow flexion, wrist flexion/extension) in ten hemiplegic patients.^60^ Using mobile camera recordings (POCO ×3, 1280 × 720 resolution), the research employed MediaPipe's BlazePose and BlazeHand models for anatomical landmark detection, achieving mean bias values of −0.110 to −0.540 degrees with 95% agreement limits against goniometer validation through Bland–Altman analysis. The algorithm demonstrated superior performance over existing methods (OpenPose, PoseNet, MoveNet) with enhanced accuracy, reduced computational complexity and 33 anatomical key points. Despite limitations including accuracy degradation with bone deformities and clothing interference, this cost-effective approach enables reliable movement assessment for clinical and home-based rehabilitation, facilitating real-time progress tracking and planned smartphone application development for stroke patient care.

The research demonstrates that utilizing a combination of open-source markerless motion capture pipelines, specifically MediaPipe and Anipose, can facilitate efficient data collection while maintaining accuracy in kinematic measurements for simple single finger flexion-extension movements.^61^ The study, conducted with 20 participants (reduced to 19 for final analysis), found that the number of cameras employed significantly impacts the accuracy of joint angle measurements, with differences of up to 20° observed when comparing configurations of two or three cameras against a four-camera setup. However, certain two-camera orientations, specifically using outer cameras positioned at the extremes of the capture volume, yielded results with minimal discrepancies (less than 4° over a 150–200° ROM) compared to the four-camera configuration, indicating that optimal camera placement can achieve comparable tracking performance. This approach offers substantial advantages over traditional marker-based systems that require up to 96 reflective markers that are “arduous to place and encumber participant movements.” The authors have made their methodology accessible by providing open-source code (https://github.com/DanMulla/handmocap) to reduce barriers for other researchers. However, the study was limited to simple finger movements without validation against gold-standard marker-based systems and results may not generalize to complex hand behaviors or hand-object interactions. This research underscores the potential of markerless systems to streamline hand motion analysis, thereby enhancing the feasibility of biomechanical studies and applications in rehabilitation and human–computer interaction. The implications of these findings advocate for further exploration into the generalizability of markerless tracking across diverse populations and movement types, as well as the need for inclusive measurement tools that account for variations in skin tone and validation against established motion capture standards. Overall, the study contributes to the growing body of literature advocating for the adoption of markerless technologies in biomechanical research, paving the way for more accessible and efficient methodologies in the field.

This study investigates the effectiveness of two markerless approaches, specifically the LMC (using three devices) and Google MediaPipe API (with four webcams and novel triangulation algorithms), in comparison to a traditional marker-based method for analyzing hand movements.^62^ The study involved fifteen healthy participants performing five distinct hand movements (static positioning, piano-like sequential movements, full-hand flexion, finger extension, and ball manipulation) while being recorded by all three methods simultaneously. The study developed a camera subset selection algorithm to optimize 3D reconstruction by fusing data from multiple recording devices. The captured data were analyzed using a skeletal model through the inverse kinematics method in OpenSim software, allowing for detailed comparison of accuracy via RMSEs of finger segment angles. Results indicated that the MediaPipe-based setup outperformed the LMC, achieving an average RMSE of 10.9° compared to 14.7°, with Leap Motion also showing reduced ROM compared to other methods. The markerless systems demonstrated significant cost advantages (20–200 *per device vs.*10,000–100,000 + for traditional systems) and automated processing capabilities, suggesting promising applications for markerless methods in clinical settings. However, the study also highlighted limitations, such as the sensitivity of markerless methods to camera orientation, challenges posed by occlusions, joint-specific error variations, and setup time differences between methods, which could affect the reliability of measurements. Overall, this research contributes valuable insights into the ongoing development of motion capture technologies for upper limb rehabilitation assessment. The study examines the use of a Kinect depth sensor and the MediaPipe architecture for HPE in real-time humanoid arm positioning using a custom 3D-printed robot prototype controlled via Python–Arduino serial communication.^63^ For seamless human–robot interaction, the objective is to track and estimate important body joints in changing situations. The researchers determined the joint angles required to regulate the robot's neck, shoulder, and elbow movements using an inverse kinematics approach to analyze five specific joint movements: left/right shoulder-elbow, left/right elbow-wrist, and head positioning with millisecond-level real-time communication. The fact that MediaPipe performed better than Kinect in joint angle estimation with lower error rates (standard errors of 3.42 vs. 3.72 for right elbow-wrist, 0.44 vs. 0.79 for head angles), indicating that it is a promising tool for more precise real-time applications. Overall, the study demonstrates that integrating these technologies can greatly improve humanoid robot performance and responsiveness, particularly in dynamic, cooperative environments, with promising applications in medical rehabilitation, elderly assistance, and gesture-based control systems. The study lacks discussion of generalizability beyond controlled laboratory conditions, doesn’t address scalability to diverse user populations or complex real-world scenarios and doesn’t fully address challenges with lighting sensitivity, environmental variations, and Kinect sensor misclassifications in dynamic settings.

An intelligent hand-assisted diagnosis system is presented, which aims to improve the accuracy and accessibility of hand function evaluations. The device evaluates posture and hand motions in real time by combining cutting-edge technologies such as visual perception and cognitive interaction, utilizing a monocular camera setup with deep learning–based pose estimation enhanced by YOLO algorithm and measuring joint angles across 21 anatomical landmarks for MCP, PIP, and DIP joints.^64^ The system provides useful information for clinical evaluations and treatment planning, demonstrating average prediction errors of 3.97° for extended hand positions and 9.0° for flexed positions, though limitations persist particularly in thumb DIP joint estimation (up to 29.7° error) due to occlusion challenges. It includes a comprehensive knowledge graph of hand diseases containing 4494 entities and 20,828 relationships, along with a voice-based question-and-answer feature using CNN-CTC speech recognition methods, making it easier for patients, even those with limited hand mobility, to interact with the system. Testing with nine healthy subjects using traditional goniometer validation has shown it to be a reliable and cost-effective alternative to expensive wearable sensor systems for diagnosing hand-related conditions and aiding poststroke rehabilitation. Looking ahead, the system aims to incorporate large-scale language models and work closely with clinical teams to bring this technology into real-world healthcare applications, ultimately improving outcomes for patients with hand function impairments.

Hemiparesis, often resulting from central nervous system disorders such as stroke and cerebral palsy, presents significant challenges for patients in assessing their functional states, leading to inadequate rehabilitation. Traditional methods like the FMA are effective but time-intensive, requiring 20–30 min per assessment and specialized training, limiting accessibility in resource-constrained settings, prompting recent advancements in automated assessments using pose estimation techniques and depth cameras. Studies have shown the potential of devices such as Kinect and MediaPipe frameworks for generating joint coordinate matrices and extracting kinematic features for ML model training using XGBoost algorithms with two-step binary classification and fivefold cross-validation methodology. In validation studies with 94 patients, automated FMA systems, incorporating depth cameras and force sensors, demonstrate good validity with sensitivity and specificity averages of 0.69 and 0.84, achieving correlation coefficients of 0.960 for total scores and outperforming video-based manual assessment in most contactless items.^65^ Linear regression models and statistical analyses indicate high accuracy in automated assessments, with better agreement with face-to-face evaluations compared to manual video-based methods, requiring specific hardware setup with camera positioning at 30° depression and caregiver assistance for force-dependent tasks. Although automated assessments reduce clinician workload and enhance compliance, enabling telerehabilitation and remote monitoring capabilities with 76.6% of patients completing assessments independently, limitations include the underrepresentation of mild cases and challenges in grasp strength measurement. These systems offer standardized, objective assessments that could improve healthcare accessibility in developing countries. Future research with larger sample sizes could further validate these systems, emphasizing the feasibility of ML and depth camera technologies in streamlining rehabilitation assessments.

The survey presents advancements in markerless motion capture, DL and AI for the assessment of upper limb and motor functions, with a specific focus on rehabilitation applications. Techniques including Kinect-based systems, LMCs, and Mediapipe-enabled models offer economical and accessible solutions suitable for both clinical and home environments. The methods exhibit a high level of accuracy in applications such as gait analysis, finger movement recovery, and proprioception evaluation. Common limitations include the presence of small datasets, a focus on static tasks and restricted validation within diverse or clinical populations. Future work must address these gaps by utilizing larger and more diverse samples, implementing dynamic task evaluations, and incorporating real-time capabilities to improve applicability in real-world scenarios. Tables 2 and 3 present the comparative analysis. As evident from the Population column in Tables 1–3, the majority of reviewed studies were conducted on healthy participants, whereas only a smaller subset involved stroke-specific cohorts. This observation highlights a continuing imbalance in clinical validation, limiting the ecological validity of current markerless motion-capture systems.

**Table 2.** Comparison of advanced markerless motion capture and AI-based assessment systems for rehabilitation and motor function analysis.

Ref	System used	ML method	Pose estimation	Gesture/Task		Population	Parameter assessed	Strengths	Limitations	Application
^39^	Kinect Depth Camera	ANN	Inbuilt (31 joints)	13 FMA tasks		41 Stroke Patients	Shoulder, elbow angle	High correlation; cost-effective	Limited FMA coverage; occlusion	Stroke rehab assessment
^40^	Monocular Camera	CNN	MediaPipe^33^, HybrIK^29^	Yoga, biking, walking		Open stroke	Multiple joints + smoothness	69% cost reduction via outlier correction	Lighting, occlusion issues	Rehab
^41^	RGBD + AI	CNN	Stacked hour-glass	Arm ROM tasks		30 Healthy	Shoulder angles	Bias *<*10°; Excellent ICC	Lower accuracy in ER; std. issues	Clinical evaluation
^42^	Webcam	CNN	MediaPipe	Finger-point gesture		16 Stroke, 40 Healthy	Finger joints	Affordable, user-friendly	Focused only on fingers	Clinical application
^43^	Egocentric Head Cam	Deep learning	Hand object detector	Daily activity		24 Stroke Survivors	Hand use ratio	High clinical correlation	Manual annotation limits scale	Rehab assessment
^44^	Kinect v1	SSVM	Custom Pose Estimation	Cooking task		Cooking Action Dataset	Trajectory focus	Accurate object tracking, dynamic detail	Data quality dependency	Activity recognition
^45^	Wyze Cam v2	LSTM	HRNet	Natural task		23 Poststroke	Shoulder, elbow angles	Robust model; high accuracy	Only left arm included	Upper limb function
^46^	GoPro Hero 7	ANN	DeepLabCut	Nine-Hole Peg Test		31 patients, 58 control	22 kinematic params	High disease classification accuracy	Real-time constraints; clinical validation needed	Manual Dexterity Assessment
^47^	Retrospective Study	CART, KNN, LR, SVM	Traditional Scores	Box, WMFT tasks		30 + 48 Stroke	FMA, WMFT scores	Key predictor identification	Older age group bias	Arm nonuse prediction
^48^	Kinarm Exoskeleton	DNN, SVM, LR, DT, RFT	Robotic sensors	Position matching		429 stroke, 465 control	Variability measures	Wide ML coverage	Single task only	Position matching
^49^	Kinect + Algorithm	PCA	Inbuilt Kinect	10 UE movements		9 stroke	Shoulder, elbow, wrist	Home therapy potential	Small sample size	Clinical assessment
^50^	Clinical Scores	RF + XAI	Traditional	FMA tasks		95 stroke	ADL activity	High performance metrics	Medium sample size	Stroke rehab
^51^	2 RGB Cameras	KNN, DT	OpenPose	Reaching tasks		10 stroke	Trunk, shoulder, elbow	Targets compensatory movement	Small dataset	Clinical assessment
^52^	Leap Motion Controller	Nonlinear SVM	Inbuilt LMC	Finger flex/ext		24 stroke	Peak angle, velocity	Objective hand quantification	Low clinical applicability	Stroke rehab

ADL: activities of daily living; FMA: Fugl–Meyer Motor Assessment; MMF: Mixed-Matrix Factorization; PCA: Principal Component Analysis; XAI: Explainable AI.

**Table 3.** Comparison of advanced markerless motion capture and AI-Based assessment systems for rehabilitation and motor function analysis continued.

Ref	System used	ML method	Pose estimation	Gesture/Task	Population	Parameter assessed	Strengths	Limitations	Application
^53^	Commodity Camera	Deep learning	OpenPose	Walking	1026 CP patients	Gait analysis	Low-cost alternative to MoCap	Single-plane view only	Remote Monitoring
^54^	Kinect v2	Deep learning	Inbuilt Pose	Daily tasks (e.g., combing hair)	13 Healthy Male	Shoulder, elbow movement	High CMCs >0.93; low cost	Occlusion sensitivity; limited accuracy	Home Rehab
^55^	Azure Kinect RGB-D	Deep learning	MediaPipe	Fine hand motions	27 Stroke Patients	Finger joint movement	Enhanced accuracy	Limited generalizability	Stroke Rehab
^56^	Wearable IMU + Video	Ensemble ML	Custom algorithm	Upper limb ADLs	20 Stroke, 20 Healthy	Joint angles + motion energy	Multimodal integration	Sensor setup complexity	Rehab Monitoring
^57^	Smartphone Camera	CNN-LSTM	BlazePose	Sit-to-stand task	35 Stroke	Lower limb kinematics	Easily deployable tech	Model training needs personalization	Mobility Monitoring
^58^	Stereo Camera System	SVM + RF	Custom Skeleton Model	Upper body tasks	15 Stroke Patients	Movement smoothness	High 3D capture fidelity	Costly setup	Motor Function Evaluation
^59^	RealSense RGBD Cam	CNN	OpenPose + SkeletonFit	Sit-to-walk transitions	32 Stroke Patients	Trunk + limb movement	Realistic task simulation	Poor generalization to varied setups	Rehab Progress Tracking
^60^	Kinect + Accelerometer	Decision Tree	Hybrid system	Arm lift + reach	14 Stroke Survivors	Angular velocity + amplitude	Sensor fusion improves accuracy	Small cohort	Arm Movement Evaluation
^61^	Video from web cam	deeplearning	Mediapipe	Finger flexion Extension	19 healthy person	Finger Joint angle	optimal placement of just 2 cameras can achieve similar accuracy to 4 cameras	only applicable to simple single finger flexion-extension movements	poststroke assessment
^62^	Smartphone RGB Video	ML Ensemble	PoseNet	Gait	60 CP Children	Gait phase and stride	Clinical utility with mobile tech	Requires ideal lighting	Pediatric Gait Analysis
^63^	WebCam	Deep learning	DeepPose	Seated balance task	30 Stroke Patients	Trunk sway angles	Real-time balance tracking	Limited to sitting posture	Trunk Rehab
^64^	Action Camera	CNN + RNN	BlazePose	Hand washing task	15 Elderly	Arm reach, timing	Hygiene + motor monitoring	Camera placement critical	Elderly ADL Monitoring
^65^	Azure kinect	XGBoost	Mediapipe	Synergy Movements	94 stroke Patients	Joint angles during movements	Largest study with high accuracy	Poor grasp assessment validity	poststroke assessment
^66^	RGB Camera + Web App	CNN-LSTM	MediaPipe + LSTM	Brush hair, reach object	25 Stroke Patients	Motion path + joint timing	Home-based monitoring viable	Dependent on app compliance	Remote Functional Assessment

To provide a standardized comparison, Table 4 summarizes key markerless motion-capture systems for upper-limb assessment after stroke, based on the studies included in this review. The comparison highlights accuracy, validation type, and real-time capability under consistent criteria. Overall, AI-based frameworks such as Theia3D and OpenPose demonstrate higher accuracy and adaptability than earlier Kinect-based systems, although large-scale clinical validation remains limited.

**Table 4.** Comparative summary of markerless motion-capture systems for upper-limb functional assessment after stroke.

System/Framework	Representative studies	RMSE/Error range	ICC/Reliability	Real-time capability	Clinical validation	Accuracy summary	Key remarks
Kinect-based systems	(18; 21; 28; 30; 33; 34; 36; 49; 54; 65)	6–16°	Up to 0.93	Partial (∼33 ms)	Healthy and stroke (*n* = 10–94)	Accurate for proximal joints; limited distal fidelity	Low-cost and simple setup; affected by occlusion and lighting.
OpenPose-based systems	(20; 27; 29; 51)	≈20–30 mm (MAE)	*>*0.9	Yes (*>*30 fps)	Stroke + healthy (*n* = 10–25)	Reliable for 2D/3D pose estimation	Requires multiple cameras; accuracy depends on lighting and viewpoint.
MediaPipe frameworks	(40; 42; 57; 59; 60; 61; 62; 63)	5–10°	0.85–0.92	Yes (>30 fps)	Mainly healthy (*n* = 10–40)	Good accuracy for simple limb movements	Lightweight and mobile; limited validation in stroke populations.
Theia3D	^24^	*<*6°	0.7–0.9	Yes	Children (upper extremity; Box and Blocks test)	Comparable reliability to marker-based; small bias observed	Preliminary validation; limited to UE motion in children.
Leap Motion Controller	(32; 38; 52)	<15 mm	—	Yes	Stroke + healthy (*n* = 13–44)	Precise for finger/hand tracking	High spatial fidelity; limited range and lighting sensitivity.
Hybrid/Multimodal systems	(23; 30; 41; 58)	3.7–7.3°	Up to 0.99	Partial (depends on fusion)	Healthy (*n* = 13–82; rehab-oriented)	Robust multimodal accuracy	High computation; setup complexity.

MAE: mean absolute error; RMSE:root mean square error.

### Visual summary of markerless system usage

**Figure 6.**
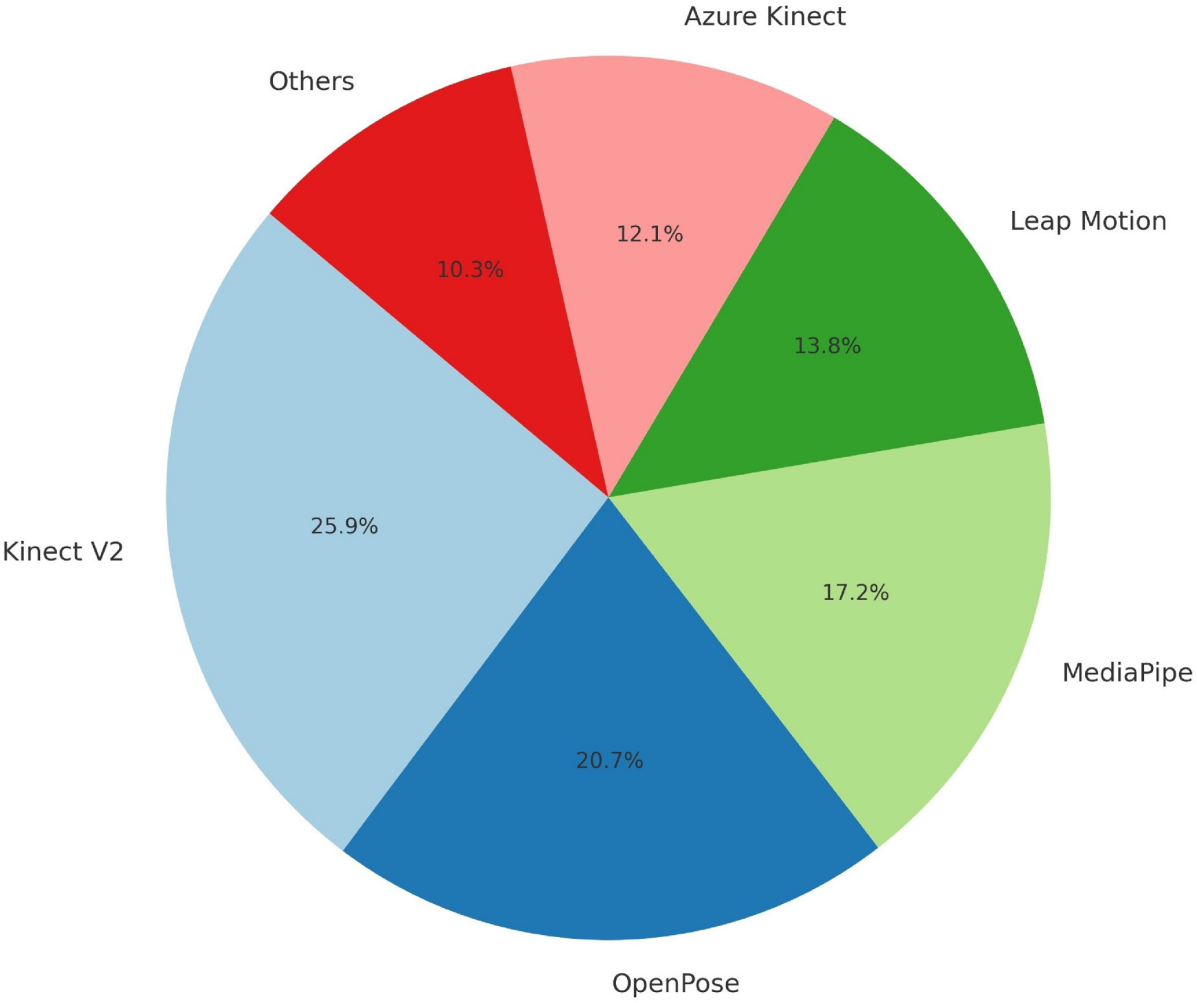
Visual summary of markerless system usage.

To enhance clarity regarding the technologies used across the reviewed literature, Figure 6 presents a visual summary of the distribution of markerless motion capture systems. The figure aggregates data from both technical system evaluations and AI-integrated assessments. As depicted, Kinect V2 and OpenPose were the most widely employed platforms, followed by MediaPipe, Leap Motion, and Azure Kinect. This reflects both the accessibility and maturity of these tools in stroke rehabilitation research. The chart also highlights a reliance on a small number of platforms, suggesting the need for broader validation of alternative or emerging systems.

### Validation methods across reviewed studies

**Figure 7.**
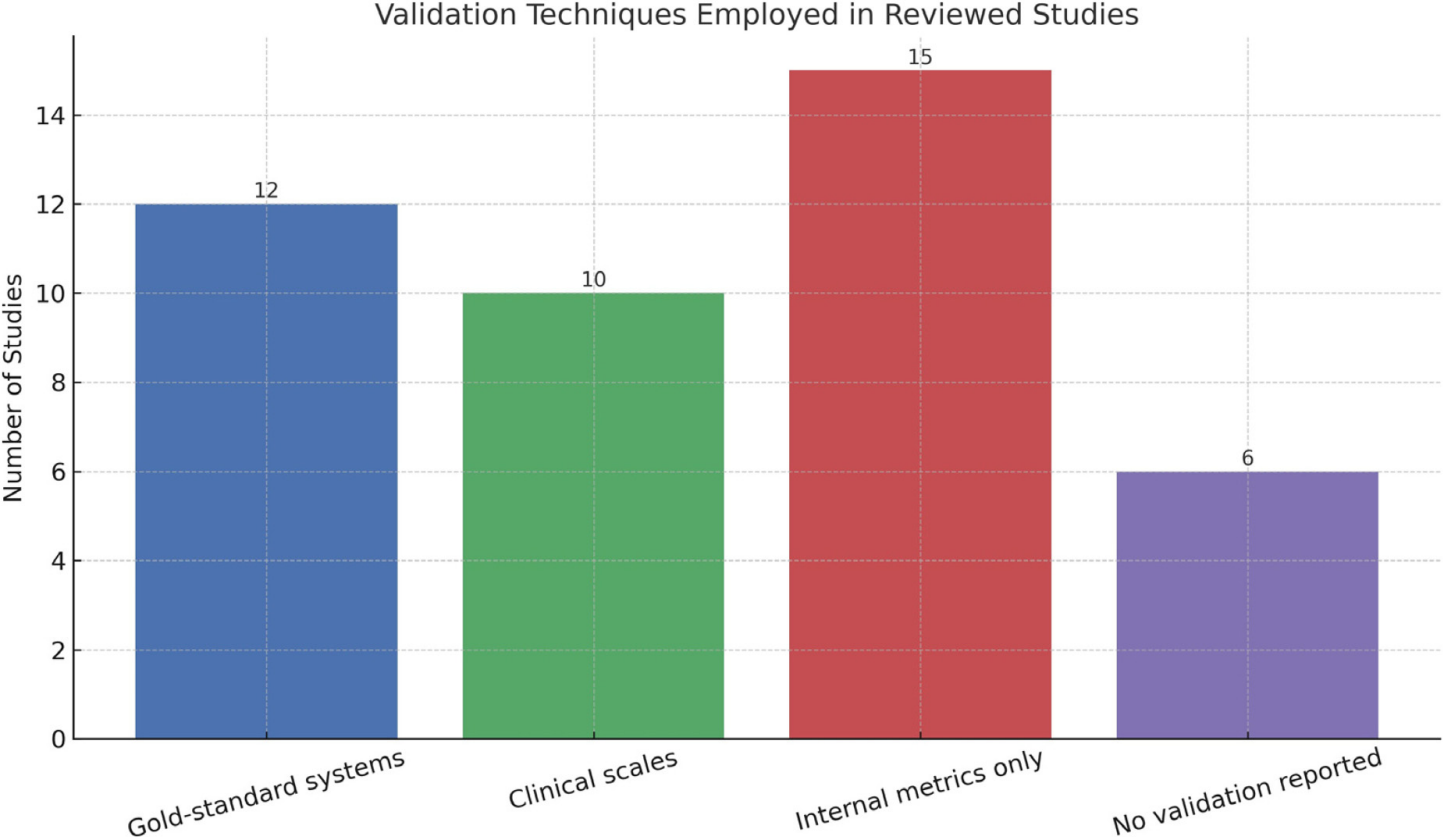
Validation methods across reviewed studies.

To assess the methodological rigor of the reviewed studies, Figure 7 provides a breakdown of the validation methods employed. While many studies utilized internal metrics such as RMSE, MAE, or correlation coefficients, fewer incorporated validation against clinical scales (e.g., Fugl–Meyer Assessment, WMFT) or gold-standard systems such as Vicon or goniometry. Notably, a portion of the studies did not report any formal validation method. This visualization highlights a key limitation in the field: although technical feasibility is often demonstrated, clinical robustness, and external benchmarking are inconsistently applied. These trends reinforce the importance of standardized and clinically meaningful validation in future research.

### Machine learning model usage across application domains

**Figure 8.**
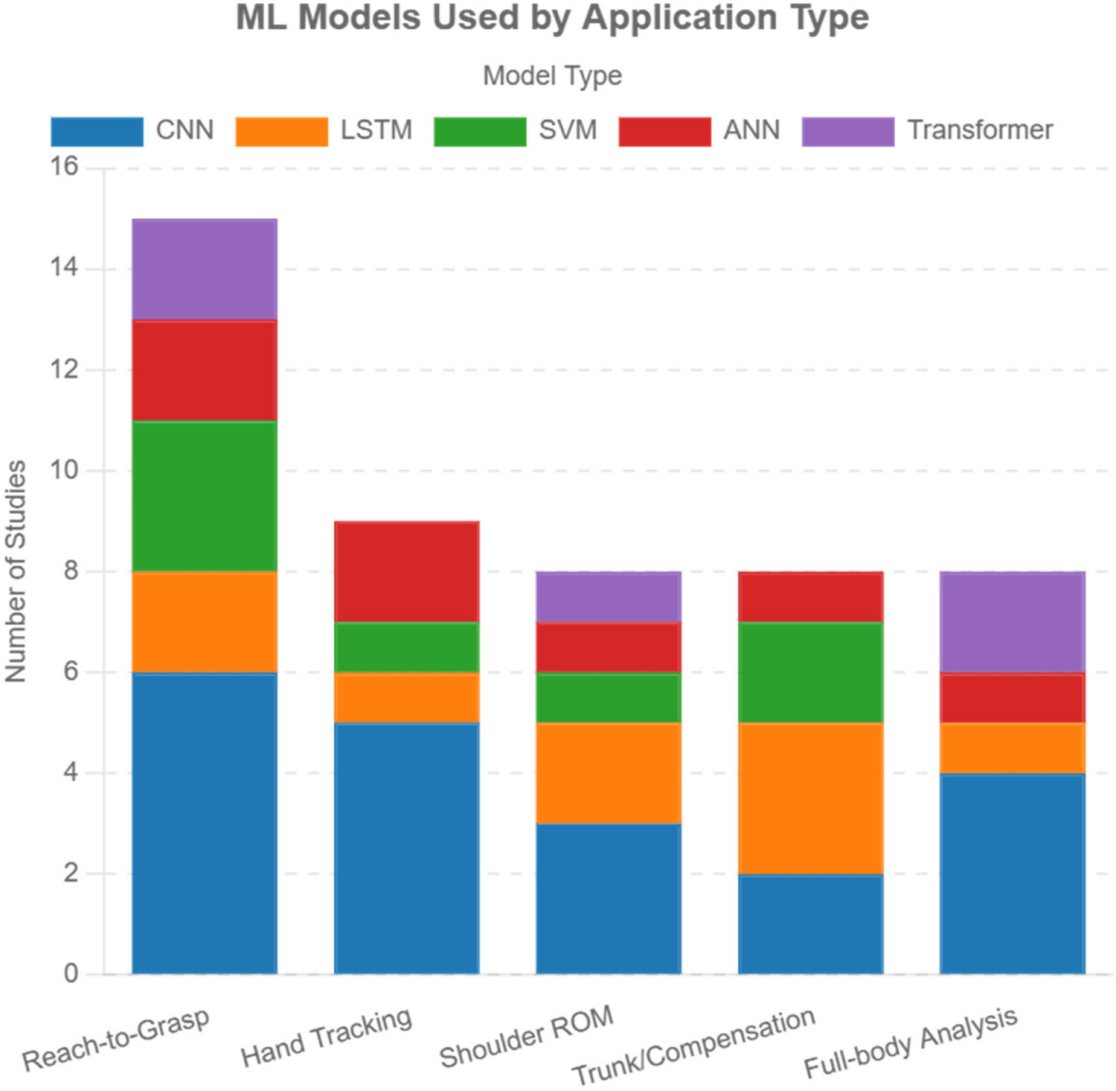
Ml model usage across application domains.

Figure 8 illustrates the distribution of ML models across various application areas in poststroke rehabilitation, such as reach-to-grasp tasks, hand tracking, and shoulder range-of-motion assessments. CNNs were the most frequently used models across all domains, especially in hand tracking and reach-to-grasp applications. LSTM and SVM models were more commonly used in tasks requiring temporal pattern recognition or compensation detection. The chart highlights how model selection is often driven by task complexity, data type, and motion dynamics. This visualization supports the need for task-specific model optimization and standardization in future work.

### Appraisal of AI and ML-based approaches

Table 2 and 3 outlines the technical architectures, validation methods, and clinical relevance of AI-enabled markerless motion capture systems. While deep learning models such as CNNs, LSTMs, and Transformers achieved high classification accuracy (often *>*90%) and strong agreement with clinical scores (e.g., FMA, WMFT), a critical evaluation reveals several limitations. Many studies relied on small, homogeneous datasets, often collected under controlled lab conditions with healthy individuals instead of actual stroke patients. This raises concerns regarding generalizability and model robustness.

Moreover, few studies included rigorous external validation, long-term follow-up, or assessment under real-time conditions. Tasks were typically static or scripted, limiting the ability to assess compensatory movements or natural variability in patient behavior. While performance metrics such as F1-score and AUC were often reported, interpretability and clinical integration remain challenges—especially for black-box models without XAI frameworks.

Despite these limitations, the reviewed ML approaches demonstrate strong potential for automating motor function evaluation and enabling personalized rehabilitation. Future research should focus on larger, diverse clinical populations, unscripted task analysis, and real-time feedback mechanisms to ensure safe and effective deployment in clinical and home-based settings.

## Challenges and opportunities

The following discussion highlights current challenges and practical considerations for clinical implementation. To facilitate the widespread adoption of markerless motion capture systems in clinical and telerehabilitation environments, several critical challenges must be addressed particularly those unique to poststroke upper limb assessment.

Current systems often demonstrate limited generalizability, as they are frequently trained and validated on small, homogeneous datasets, typically involving healthy participants. This constrains their effectiveness when applied to stroke patients who exhibit diverse and complex motor impairments. A substantial proportion of studies were conducted on healthy participants under laboratory conditions. Only a minority evaluated stroke-specific populations, often with small sample sizes. This limits clinical generalizability and underscores the need for patient-specific benchmarking and validation in real-world rehabilitation contexts. As summarized in Tables 1, 2 and 3, this imbalance is consistent across most categories of markerless systems, clearly delineating healthy subject validation from clinically oriented studies. The present review explicitly critiques this disparity, emphasizing that healthy subject validation while technically informative cannot substitute for clinical population testing. The limited number of stroke specific validation studies restricts clinical generalizability and calls for standardized patient centric benchmarking across diverse rehabilitation contexts.

Moreover, environmental variability, such as changes in lighting, background clutter, and frequent occlusions, significantly impacts the robustness and reliability of these systems in real-world or home-based settings. Another major limitation is the lack of real-time processing in many approaches, which hinders the implementation of interactive and adaptive rehabilitation protocols. Real-time implementation remains constrained by computational latency, camera synchronization, and data transfer bottlenecks. Lightweight pose-estimation models and on device (edge) computing could mitigate these barriers, enabling responsive feedback critical for home-based rehabilitation.

A notable gap in the literature is the lack of comprehensive upper limb evaluations. Most existing studies concentrate on isolated joints such as the wrist or shoulder while overlooking the coordinated function of the entire upper limb. Additionally, occlusions during overlapping or complex movements, along with insufficient patient specific calibration, present further challenges to accurate joint tracking and motion analysis.

Despite these constraints, recent advancements offer promising opportunities. The integration of deep learning techniques with multimodal sensor inputs (e.g., RGB-D cameras) can significantly improve tracking accuracy, adaptability, and resilience in uncontrolled environments. The development of cost-effective, scalable systems holds promise for extending access to personalized rehabilitation in underresourced settings. Furthermore, coupling real-time feedback mechanisms with cloud-based analytics can enable dynamic, adaptive therapy, and continuous remote monitoring.

Overcoming these challenges is essential to transition markerless motion capture systems from experimental tools to clinically validated solutions, with the potential to transform poststroke rehabilitation and significantly improve patient outcomes.

## Conclusion

This review highlights the significant potential of markerless, video-based motion capture systems in the functional assessment of upper limbs for poststroke rehabilitation. By synthesizing findings across recent studies, we identified consistent trends in the use of depth cameras, pose estimation algorithms and ML models to analyze joint kinematics such as shoulder, elbow, and wrist movements. While these technologies offer promising advantages such as noninvasiveness, real-time capability, and adaptability to home-based care, key challenges remain. These include limited dataset diversity, difficulty in capturing complex or occluded movements, and a lack of comprehensive systems that evaluate the entire upper limb. Moreover, a critical appraisal of the reviewed studies revealed variability in methodological quality, including the use of small sample sizes, healthy participants instead of clinical populations, and inconsistent validation procedures. These limitations affect the generalizability of results and highlight the need for standardized, clinically validated approaches. To enable wider clinical adoption, future work should focus on enhancing robustness in real-world settings, improving model generalization through diverse training datasets and developing end-to-end systems capable of supporting adaptive and remote rehabilitation strategies. Overall, markerless motion capture presents a viable and scalable approach for improving poststroke assessment and personalized therapy, especially in resource-constrained and telerehabilitation contexts. Markerless motion capture offers an accessible, objective, and scalable solution for upper-limb assessment after stroke. Future research should emphasize standardized validation and clinical integration to enable widespread implementation in rehabilitation practice. Additionally, prioritizing the development of standardized open datasets, cross-platform benchmarking protocols, and integration with electronic medical record platforms will help accelerate translation of markerless motion capture from research to clinical use.
